# The Biocurator: Connecting and Enhancing Scientific Data

**DOI:** 10.1371/journal.pcbi.0020125

**Published:** 2006-10-27

**Authors:** Nima Salimi, Randi Vita

**Affiliations:** National Center for Biotechnology Information, United States of America

From Impressionism and Pop Art to phosphorylation sites and interacting atom pairs, the realm of curation has been expanded. The recent growth of bioinformatics, driven by exponentially growing data, advanced computing techniques, and increased funding from private and governmental organizations, has created the need for novel strategies to adequately capture, store, and analyze the multitude of data present in the scientific literature. To meet this challenge, the number and scope of scientific databases has soared in recent years, creating a new profession, the biocurator. Indeed, the present emphasis on expanding computational resources, capable of managing and analyzing complex biological data, presents an ever-growing demand for biocurators capable of interpreting the increasingly complex scientific literature and extracting relevant data in an efficient, yet consistent, manner.

The Immune Epitope Database and Analysis Resource (IEDB) at http://www.immuneepitope.org [1,2] was established to capture, house, and analyze complex immune-epitope–related data extracted from the scientific literature by a team of specialized biocurators. Our experiences as IEDB biocurators are presented here to provide insight into the role of the biocurator and the challenges of literature-based curation of complex scientific data.

The goal of the IEDB is to provide the scientific community with open access to concise and comprehensive immunological data and analysis resources in a previously unavailable format. The IEDB catalogues epitope sequences and structures; however, we further expand the magnitude of accessible information by including data regarding the immunological contexts in which the epitopes are defined and assayed (MHC binding, T cell, B cell, or MHC ligand elution). This affords the user the ability to generate refined queries to selectively access data of interest. To achieve this utility, our biocurators manually capture immunological data from the published literature at an unprecedented level of detail that includes data fields ranging from simple concepts such as the antigen, immunogen, and assay type to more advanced fields such as the TCR chain types, TCR residues interacting with the epitope MHC complex, and detailed information regarding carriers or vectors. Therefore, interpretation of the highly detailed and complex experimental data included in the IEDB requires a team of graduate-level biocurators with both theoretical and research experience in immunology and related fields. The IEDB currently employs eight full-time and two part-time scientists as biocurators.

Although IEDB biocurator duties are diverse, their primary role is curation of data from the published literature. The initial curation of a typical manuscript requires approximately four hours, reflective of the high degree of detail that is captured from each reference (published article). While the granularity of the curated data distinguishes the IEDB as a novel resource, it also necessitates specific curation guidelines and a comprehensive review process that ensures accuracy and precision of each curation prior to its release into the public database. The IEDB biocurator plays a key role both in the formulation of these guidelines and in the review process.

The nature of the data relevant to the IEDB required us to establish well-defined curation guidelines to promote consistency and to clearly delineate objective *representation* of the data from subjective *interpretation* of the data. In conjunction with a group of prominent senior immunologists, known as the Epitope Council (EC), the biocurators continuously develop the *Curation Manual*. This manual provides precise instructions regarding the strategies and procedures for capturing, annotating, and introducing complex and detailed data from the literature into the IEDB. The *Curation Manual* is used to ensure validity, standardization, and the efficiency of the curation process, and coevolves with the database as we continually encounter circumstances that require new guidelines to be established. The current *IEDB Curation Manual* (version 14) is publicly available through the IEDB website.

Despite the use of our extensive *Curation Manual,* there are difficult situations that inherently arise during curation. We often encounter inconsistent terminologies in the literature that present formidable challenges to our consistent interpretation of the data. Scientists frequently use highly diverse and controversial nomenclature, for example, in the naming of MHC molecules. The methods used to perform an experiment may be somewhat obscure or contradictory. The conclusions drawn by the authors may be difficult to represent based upon the limitations of the database fields and our curation guidelines. Newly created assay types may require interpretation and assignment to a particular assay group. Thus, valuable meetings involving the curation team and the EC are held weekly to discuss novel issues arising in curation and to review specific references. While every effort is made to address such problems using our established guidelines, novel challenges are often dealt with on a reference-by-reference basis. The solution is then translated into a generalized guideline that can be consistently applied to similar occurrences in the future.

The level of detail and precision that we aspire to capture can lead to disagreements over the specifics. Issues as simple as assigning an effector cell type, the semantics used to describe an assay type, or even the qualitative result can lead to a great deal of discussion. Many times authors may discuss conclusions or assumptions that are not depicted in the figures. As the goal of the IEDB is to present as much information as possible without subjective interpretation, we can never presume any information, but rather we must try to capture the data exactly as presented in the reference, while maintaining the conclusions of the reference in a uniform manner. For example, if all experiments are performed with a whole cell population, but the authors attribute the response to a particular cell type without any evidence, we must capture the effector cells as the entire population.

To further ensure accuracy, we often contact the authors of papers to clarify details of the experimental procedures, to obtain epitope sequence data, or to request specific information regarding the source of the epitope. For example, we strive to link each curated epitope to an appropriate GenBank or SwissProt database entry in order to provide the user with the exact source of the published epitope. Often, this valuable link cannot be assigned due to an amino acid discrepancy between the sequence provided in the manuscript and the sequences present in GenBank or SwissProt. In these cases, we attempt to resolve this issue by contacting the author or searching the citations in the manuscript. In our experience, these efforts result in successful contact and clarification in approximately 50% of cases and serve to enhance a significant amount of our data. Additionally, authors may provide feedback, corrections, or clarification to any data present within the IEDB through links provided on the Web site. In the event of a disagreement, the biocurator would work with the author to rectify the data representation. However, to this date, no such conflict has arisen.

For further quality assurance, we have implemented a two-tiered review system, consisting of an initial phase of peer review, followed by detailed review by the EC. Senior biocurators typically spend 25%–50% of their time peer-reviewing the curations of other biocurators. This is an interactive process, in which we discuss the curation and the reviewer recommends modifications. The subsequent EC review process not only adds another layer of scrutiny, but also provides an opportunity to evaluate our curation guidelines and the scope of the database in light of specific references. This process has proven to enhance the quality of the curation that ultimately becomes public.

In addition to curation, IEDB biocurators typically devote approximately 25% of their time to non-curation efforts relating to the IEDB. This enables the biocurator to become involved in a variety of projects. As a novel database, the design and implementation of the IEDB offers many challenges that require a creative approach. Since the database has been public for less than six months, biocurators at the IEDB have the opportunity to be involved with developmental aspects of the project such as software programming, tool development, and database design, in addition to curation. For example, several biocurators are currently enhancing the IEDB ontology which will be made publicly available for use by the scientific community or relevant databases. Furthermore, certain biocurators are focusing on becoming experts in analyzing the data they curate. For example, all influenza epitopes present in the IEDB were recently analyzed for sequence conservancy between the different strains (unpublished data: Bui HH, Peters B, Assarsson E, Mbawuike I, Sette A, Antibody and T cell epitopes of Influenza A virus—Knowledge and opportunities).

Communication with authors and end-users is a critical skill for the IEDB biocurator for the purposes of curation, database development, and for community outreach efforts to raise public awareness about the IEDB. We often consult with outside scientists for subject-matter expertise. For example, because the IEDB staff is composed of scientists with predominantly T cell expertise, we have sought external B cell experts for input on database design and the development of related curation rules. IEDB biocurators also act as ambassadors for the database, both to raise community awareness and to collect positive and constructive feedback. We must be aware of the needs of the IEDB end-user to productively enhance the curation guidelines, database structure, and ontology. We attend national and international conferences to promote and discuss the database with both end-users and contributors. Representatives from the IEDB attended the First International Biocurator Meeting in December 2005 where curation teams representing 106 databases gathered to exchange information and ideas. Additionally, the IEDB was presented at both the 2006 Keystone Symposium and The Annual Meeting of American Association of Immunologists (2006).

The biocurator's role is dynamic and evolves in parallel with developments in bioinformatics, bridging the gap between knowledge and its accessibility. Regardless of the source and subject of the data, the biocurator accumulates disparate, but relevant data into one centralized location where it is made more accessible to researchers. The organized and analyzed data are thus made available for retrieval, analysis, and download by the end-user in an enhanced format, providing an added dimension of utility that would not otherwise be present.

As biocurators, we must be able to understand scientific data and to incorporate the curation guidelines in a way that maintains the integrity of both. Open communication between the scientists and the biocurators may foster a better understanding of the difficulties encountered by the biocurator and facilitate a more standardized approach to data representation in the literature. The growing use of and contribution to various high-impact databases underscores the need to establish or refine standardized biological vocabularies and definitions. With more experienced scientists as biocurators, an accurate and controlled vocabulary among database users and contributors can be developed and promoted. The use of a shared vocabulary increases curation speed, data consistency, and serves to connect the information contained in multiple databases. Endeavors to achieve uniformity significantly enhance the efficiency of data curation and exchange. Such reform should eventually diffuse throughout the scientific community and be reflected in the literature.

New methodologies allow scientific data to grow at an exponential pace, creating a steady demand for reliable, consistent, and accurate databases. As bioinformatics resources continue to grow, so will the role of the biocurator in their development. The expansion of bioinformatics and its applications paints a promising future for the biocurator. 

## Abbreviations

EC: Epitope Council
IEDB;: Immune Epitope Database and Analysis Resource (IEDB)
